# The therapeutic protection of a living and dead *Lactobacillus* strain against aluminum-induced brain and liver injuries in C57BL/6 mice

**DOI:** 10.1371/journal.pone.0175398

**Published:** 2017-04-07

**Authors:** Fengwei Tian, Leilei Yu, Qixiao Zhai, Yue Xiao, Ying Shi, Jinchi Jiang, Xiaoming Liu, Jianxin Zhao, Hao Zhang, Wei Chen

**Affiliations:** 1 State Key Laboratory of Food Science and Technology, School of Food Science and Technology, Jiangnan University, Wuxi, Jiangsu, P.R. China; 2 UK-China Joint Centre on Probiotic Bacteria, Norwich, United Kingdom; 3 Beijing Innovation Centre of Food Nutrition and Human Health, Beijing Technology & Business University, Beijing, P.R. China; Chinese Academy of Sciences, CHINA

## Abstract

Our previous study found that *Lactobacillus plantarum* CCFM639 had the ability to alleviate acute aluminum (Al) toxicity when the strain was introduced simultaneously with Al exposure. This research was designed to elucidate the therapeutic effects of living and dead *L*. *plantarum* CCFM639 against chronic Al toxicity and to gain insight into the protection modes of this strain. Animals were assigned into control, Al only, Al + living CCFM639, and Al + dead CCFM639 groups. The Al exposure model was established by drinking water for the first 4 weeks. The strain was given after Al exposure by oral gavage at 10^9^ colony-forming units once per day for 12 weeks. The results show that the Al binding ability of dead CCFM639 was similar to that of living CCFM639 *in vitro*. The ingestion of living or dead CCFM639 has similar effects on levels of Al and trace element in tissues, but living strains led to more significant amelioration of oxidative stress and improvement of memory deficits in Al-exposed mice. In conclusion, in addition to intestinal Al sequestration, CCFM639 treatment offers direct protection against chronic Al toxicity by alleviation of oxidative stress. Therefore, *L*. *plantarum* CCFM639 has a potential as dietary supplement ingredient that provides protection against Al-induced injury.

## Introduction

Aluminum (Al) exists throughout nature (in air, water, and plants) and consequently in almost all food [1]. It is also most frequently used in food technology, including packaging materials, food additives, and kitchen utensils [2]. Humans therefore cannot avoid exposure to Al. Once Al is absorbed, it can accumulate in variety of organs [3]. It can cross the blood-brain barrier and accumulate in the brain, causing degeneration of neuronal cells and affecting behavior [4, 5]. Al exposure is thus closely associated with neurodegenerative disorders, including Alzheimer’s disease, Parkinson’s disease, and dialysis encephalopathy [6]. The liver plays a significant role in contaminant storage, redistribution, and detoxification [7]. It is an important early sink for absorbed Al, and Al can be excreted through bile and finally through feces to the outside of body [8, 9], so some researches have also focused on the adverse effects of Al on the liver.

Al is not a redox active metal, but it is pro-oxidant and can induce the formation of reactive oxygen species (ROS), causing oxidative stress and cell damage in diverse tissues, including the liver and brain [10, 11]. Some lactic acid bacteria (LAB) strains have been shown to scavenge ROS and possess antioxidative ability, thus providing protection against oxidative stress and lipid peroxidation [12]. As a very important LAB genera, *Lactobacillus* has a number of human health benefits[13]. It has been widely used in fermented products that improve total antioxidant status and reduce oxidative stress in healthy individuals [14, 15]. This property may mean that *Lactobacillus* is a potentially effective tool against Al toxicity.

We recently have found that the probiotic *Lactobacillus plantarum* CCFM639 can significantly protect mice from acute Al toxicity when the strain was introduced simultaneously with Al exposure [16]. This probiotic can reduce intestinal Al absorption, decrease Al accumulation, and alleviate oxidative stress in tissues. However, it is not clear whether the alleviation of oxidative stress in tissues was simply an indirect effect of the reduction of intestinal Al absorption, a direct effect of the antioxidative property of *L*. *plantarum* CCFM639, or both. Therefore, the objective of present study was to elucidate the protections that *L*. *plantarum* CCFM639 can offer against chronic Al toxicity in mice and to gain insight into the protective mode of this strain by separating its intestinal Al sequestration capacity and identifying its other potential protective activities.

## Materials and methods

### Chemicals and kits

All of the assay kits used to measure the activities of superoxide dismutase (SOD), catalase (CAT), glutathione peroxidase (GPx), alanine transaminase (ALT), and aspartate transaminase (AST) and the levels of glutathione (GSH), malondialdehyde (MDA), blood urea nitrogen (BUN), and creatinine (CRE) were bought from Nanjing Jiancheng Bioengineering Co. Ltd. (Nanjing, China). MRS broth was bought from Hope Bio-Technology Co. Ltd. (Qingdao, China). Aluminum chloride (AlCl_3_.6H_2_O) was bought from the Sinopharm Chemical Reagent Co. Ltd. (Shanghai, China).

### The strain and preparation

*L*. *plantarum* CCFM639 (CGMCC9664) was obtained from the in-house Culture Collections of Food Microbiology (CCFM) of Jiangnan University (Wuxi, China) and cultured as previous study [16, 17]. In order to obtain living and dead CCFM639, cultured biomasses were washed with ultrapure water three times and then divided into two equal portions. One portion, the living biomass, was lyophilized with skim milk and preserved at -20°C. The other portion was boiled at 100°C for 30 min before lyophilization to obtain dead biomass. The viable quantity of biomass was measured and remained approximately at 5×10^9^ colony-forming units (CFU)/mL before it was used in the animal experiments.

### Determination of Al binding ability

The Al binding ability of *L*. *plantarum* CCFM639 was measured according to a previously used method with minor modifications [18]. The fresh living and dead strains were resuspended in ultrapure water with 5, 50, and 100 mg/L Al ion, respectively, and finally obtained 1g/L of wet bacterial concentration. The samples were incubated at 37°C for 2 h and then centrifuged at 8000 *g* for 20 min. After centrifugation, the residual Al level in the supernatant was analyzed by inductively coupled plasma mass spectrometry (ICP-MS, NexIon-300X; PerkinElmer) [19]. The background Al concentration was measured by preparing living and dead pellets in ultrapure water instead of Al solution. The final Al binding activity of the strain was assessed after deducting the background Al concentration and expressed as the Al removal rate. They were calculated using the following equation.

Removal rate = [(*C*_1_ –*C*_2_) ÷ *C*_1_] × 100% where C_1_ and C_2_ are the initial and residual Al concentration, respectively.

### Animal and experimental design

Fifty C57BL/6 mice (male, six week old) weighing 18–25 g were maintained at a controlled temperature and humidity (22°C ± 2°C, 55% ± 10%) with 12 h light/dark cycles. They were fed *ad libitum* with a standard diet and water. The animal experiments was approved by the Ethics Committee of Jiangnan University, China (JN No. 20150721-1030-51), and all procedures about the care and use of experimental animals followed the guidelines set by the European Community (directive 2010/63/EU).

After one week of adaptation period, the mice were randomly assigned to four different groups of similar mean body weight: control, Al only, Al + living CCFM639, and Al + dead CCFM639 (Table 1). The Al ion was administered in the form of AlCl_3_.6H_2_O and provided at a dose of 200 mg/L in drinking water for first four weeks (about 32 mg/kg bw/day Al ion) [20–23]. The water in feeding bottle was refreshed every week. After Al exposure, *L*. *plantarum* CCFM639 was provided for another 12 weeks at a dose of 10^9^ CFU in 0.2 mL of skim milk once daily via oral gavage.

**Table 1:** Design of the animal experiment.

Group (n = 10)	Treament	
	1–4 weeks	5–16 weeks
Control	PW	PW + SM
Al only	Al	PW + SM
Al + living 639	Al	PW + SM + living 639
Al + dead 639	Al	PW + SM + dead 639

PW, plain water for drinking; SM, 0.2 mL skim milk; Al, Al ion at 200 mg/Lin drinking water; SM + 639, 0.2 mL skim milk contained living or dead *L*. *plantarum* CCFM639 (1×10^9^ CFU once a day). Animals received skim milkand living or dead *L*. *plantarum* CCFM639 via oral gavage.

### Ethics statement

The animal experiments was carried out in accordance with the Ethics Committee of Jiangnan University, China (JN No. 20150721-1030-51), and all procedures about the care and use of experimental animals strictly followed the guidelines set by the European Community (directive 2010/63/EU). Each mouse was sacrificed by cervical dislocation with light ether anesthesia, and all efforts were made to minimize suffering.

### Sample collection and processing

During the 16-week experiment period, the body weights of the mice were measured every two weeks, and each mouse was transferred to a clean cage for 30 min every two weeks while its feces were collected. On the last day of the experiment and after fasting for 24 h, each mouse was anesthetized with ether and then sacrificed. At necropsy, blood samples were gathered and serum was stored in Eppendorf (EP) tubes. The livers and brains were carefully dissected from each animal and washed with normal saline. The serum and organ samples were preserved at -80°C until processed for following determination. It was executed on all ten mice of each group with a subdivision of serum and organs samples.

### Determination of Al and trace elements in tissues and feces

The liver, brain, and feces samples were put into metal-free digestion vessels (Omni, CEM, United Kingdom) and then digested in concentrated nitric acid with the Microwave Digestion System (MARS, CEM, United Kingdom). The levels of Al and trace elements (Fe, Mg, Zn, and Ca) in tissues and the Al level in feces were measured using ICP-MS [24].

### Determination of enzyme activities and biochemical indicators in tissues and blood

The livers and brains were assayed for SOD, GPx and CAT activity and MDA and GSH levels. The serum was assayed for ALT and AST activity and BUN and CRE levels. All of the parameters were measured with a commercially available assay kit. The experiments were performed according to the operating instructions provided by the equipment manufacturer.

### The Morris water maze test

The Morris water maze (MWM) test was performed as previously reported with minor modifications [25]. The pool was divided into 4 quadrants (I, II, III, and IV), the platform (V) was hidden in the middle of the IV quadrant. It was filled with tap water maintained at approximately 23°C. Each mouse was placed in the water facing the pool wall at a fixed position of each quadrant and allowed to swim freely to the escape platform. Mice had 4 trials per day separated by 40 min for five consecutive days (acquisition test) and permitted to find the hidden platform within 1 min. Once the mouse found the hidden platform, it would stay on it for 10 s. If the mouse could not find it in time, the experimenter would guide it toward the platform. On the sixth day (retrieval test), the platform was removed and the mouse was permitted to swim freely for 1 min. The performance and trajectory of each mouse was recorded by a camera, and the data were analyzed with the ANY-maze software.

### Determination of amyloid beta peptide levels in the brain

The levels of amyloid beta peptide (Aβ) _1–40_ and Aβ_1–42_ in the brain were measured with mice enzyme-linked immunosorbent assay kits (Cusabio, USA). The assays were performed according to instructions from the manufacturer.

### Statistical Analysis

Statistical analyses were performed using the SPSS software program, version 13.0 (SPSS Inc., Chicago, IL, USA). The experimental data were expressed as mean ± standard error of the mean (SEM). The data were analyzed by one-way analysis of variance. A probability level (*p* value) of less than 0.05 was considered statistically significant.

## Results

### The Al binding abilities of living and dead CCFM639 *in vitro*

**Fig 1:**
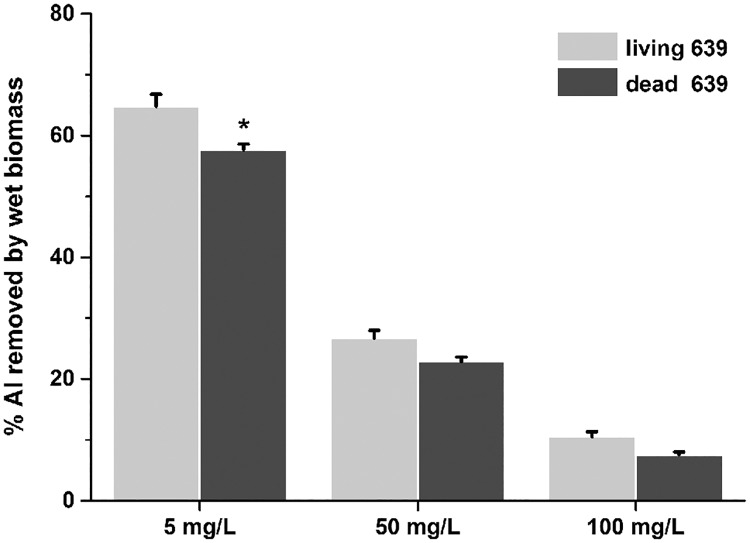
Al binding abilities of living and dead biomass *in vitro*. Data are presented as the mean ± SEM of three replicates. Asterisks on the top of the bars show significant differences between living and dead strains (*p* < 0.05).

The Al binding capacities of living and dead CCFM639 in different initial Al concentrations are shown in Fig 1. When the initial Al concentration was increased from 5 to 100 mg/L, the Al removal rate of *L*. *plantarum* CCFM639 was reduced dramatically (*p* < 0.05). Moreover, the Al binding abilities of living CCFM639 and dead CCFM639 were similar at doses of 50 and 100 mg/L Al ion, but the Al binding ability of the living strain was better than that of the dead strain at the lower Al concentration (*p*<0.05).

### Body weight

**Fig 2:**
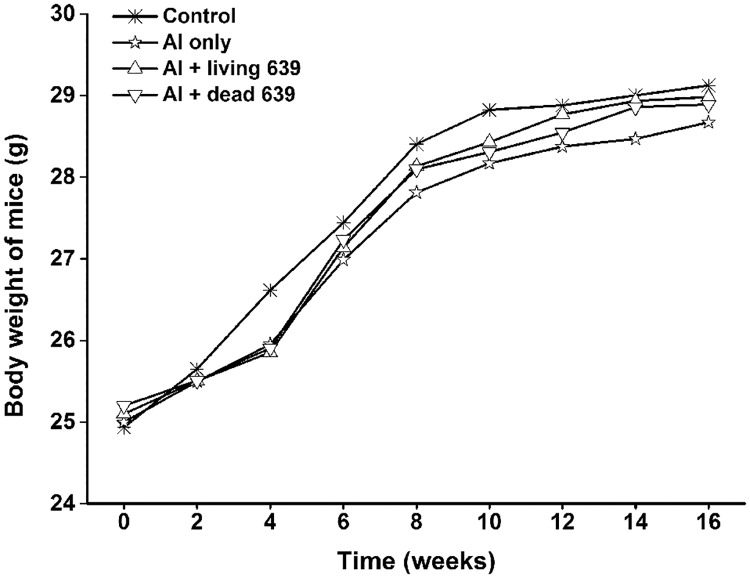
The influence of *L*. *plantarum* CCFM 639 on the body weights of mice throughout the experiment.

The body weight of all mice increased gradually throughout the experiment (Fig 2). In the first four weeks, all mice except for those in the control group grew slowly and they have a similar growth curve. In the following 12 weeks, however, the growth curves of mice in the Al plus living CCFM639 groups is the most closer to those in control group, followed by those in Al plus dead CCFM639 group and Al only group, respectively.

### Al levels in the feces

**Fig 3:**
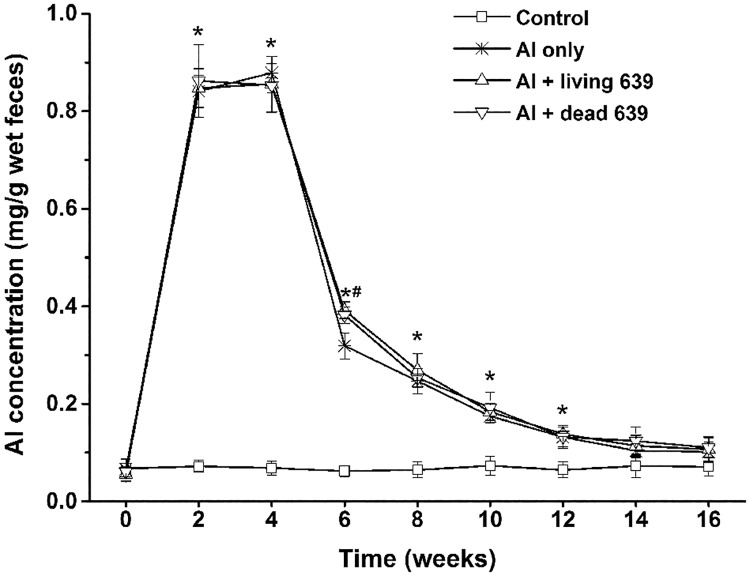
The influence of *L*. *plantarum* CCFM639 on Al concentration in the feces of mice throughout the experiment. Data are presented as the mean ± SEM of ten animals per group. Asterisks above the lines show significant differences to the control group (*p* < 0.05), and pound signs show significant differences to the Al only group (*p* < 0.05).

The changes in Al level in the feces of mice are shown in Fig 3. In the control group, the fecal Al levels were very low and remained almost constant. Compared to the control group, the fecal Al content significantly increased in other three groups during the first 4 weeks (*p* < 0.05). When exposure to Al ceased, the fecal Al content dropped dramatically. Compared with the Al only group, living and dead CCFM639 treatment significantly increased the fecal Al levels at the sixth week (*p* < 0.05), whereas in the following weeks, the *L*. *plantarum* CCFM639 strain had only a slight effect on the fecal Al levels.

### Al levels in the livers and brains

**Fig 4:**
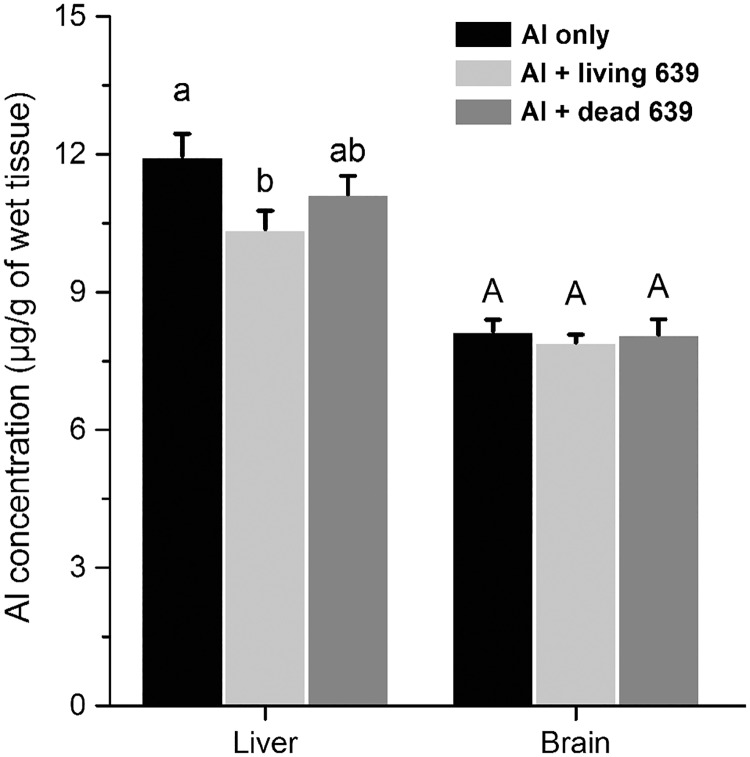
The influence of *L*. *plantarum* CCFM639 on Al concentration in the tissues of mice. Data are presented as the mean ± SEM of ten animals per group. Lowercases and uppercases on the top of the bars show significant differences among different groups (*p* < 0.05).

The Al levels in the livers and brains are shown in Fig 4. The data of Al levels in the control group are not included because the values were too low to detect. Living CCFM639 treatment significantly decreased the Al concentration in livers, but not in brains (*p* < 0.05). However, treatment with dead CCFM639 had only slight effect on the Al levels in the livers and brains compared to those in the Al-only group.

### The trace elements in liver and brain

As shown in Table 2, Al exposure significantly altered the Fe, Zn and Cu levels in the livers, as well as Fe and Zn levels in the brains (*p* < 0.05). Treatment with either living or dead CCFM639 could counter these changes, but not significantly, with the exception of the Fe level in Al plus living CCFM639 group. There was no significant difference in Cu level among the four groups in the brian, and the Ca and Mg levels among the groups in both livers and brains.

**Table 2:** The influence of *L*. *plantarum* CCFM639 on levels of trace elements in the livers and brains of mice.

Group	Mean concn (μg/g of wet tissue)				
	Fe	Ca	Zn	Mg	Cu
Liver					
Control	74.92 ± 1.48^a^	84.32 ± 2.00^a^	34.63 ± 0.89^a^	411.68 ± 12.35^a^	4.68 ± 0.16^a^
Al only	64.94 ± 1.58^b^	82.03 ± 1.68^a^	38.78 ± 1.04^b^	397.21 ± 10.03^a^	5.71 ± 0.23^b^
Al + living 639	70.84 ± 1.10^a^^b^	83.91 ± 1.66^a^	35.86 ± 1.24^a^^b^	405.35 ± 8.90^a^	5.13 ± 0.12^a^^b^
Al + dead 639	68.79 ± 1.19^a^^b^	82.92 ± 2.14^a^	36.06 ± 1.52^a^^b^	396.87 ± 6.81^a^	5.47 ± 0.11^b^
Brain					
Control	21.92 ± 0.59^a^	30.32 ± 2.37^a^	16.63 ± 0.73^a^	311.68 ± 6.78^a^	5.98 ± 0.15^a^
Al only	19.14 ± 0.47^b^	32.03 ± 2.15^a^	19.78 ± 0.76^b^	331.21 ± 8.64^a^	5.82 ± 0.20^a^
Al + living 639	21.01 ± 0.38^a^	31.91 ± 1.66^a^	17.86 ± 0.61^a^^b^	319.35 ± 8.03^a^	5.87 ± 0.24^a^
Al + dead 639	20.45 ± 0.56^a^^b^	31.92 ± 1.98^a^	18.66 ± 0.68^a^^b^	323.87 ± 5.74^a^	5.53 ± 0.27^a^

Data are mean ± SEM with ten mice in each group.

^a,b^The letters a and b indicate statistically significant differences (*p* < 0.05) among different groups.

### The GSH, MDA, SOD, CAT, and GPx in livers and brains

**Fig 5:**
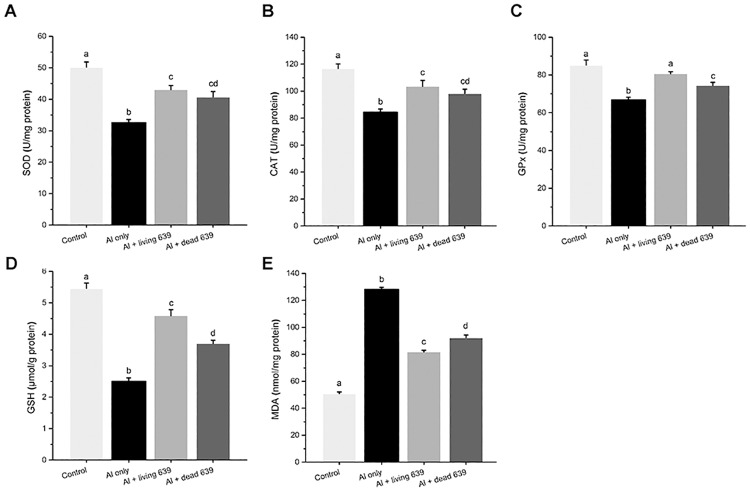
The influence of *L*. *plantarum* CCFM639 on Al-induced alterations of the enzyme activities of SOD, CAT, and GPx, and the levels of GSH and MDA in the liver. Data are mean ± SEM with ten mice in each group. The different superscript letters indicate statistically significant differences (*p* < 0.05) among different groups.

**Fig 6:**
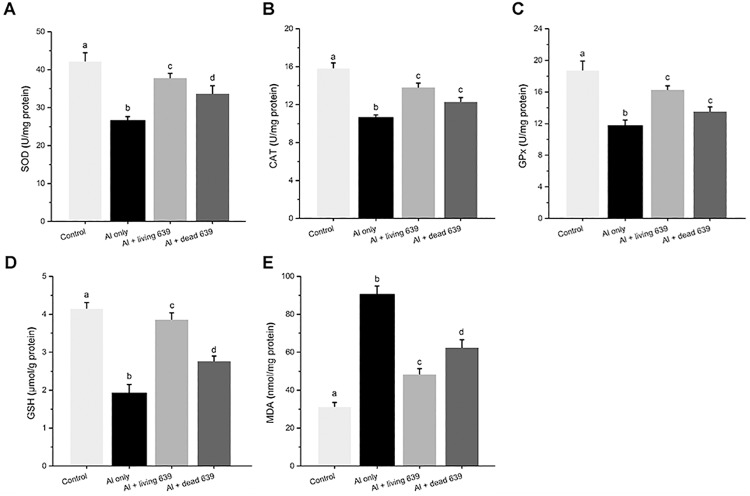
The influence of *L*. *plantarum* CCFM639 on Al-induced alterations of the enzyme activities of SOD, CAT and GPx and the levels of GSH and MDA in the brain. Data are mean ± SEM with ten mice in each group. The different superscript letters indicate statistically significant differences (*p* < 0.05) among different groups.

The changes in GSH, MDA, SOD, GPx, and CAT in livers and brains are presented in Figs 5 and 6. Compared with the control group, SOD, CAT, and GPx activity and GSH level significantly reduced in the livers and brains in the Al only group (*p* < 0.05), but markedly increased when they were treated with living or dead CCFM639 (*p* < 0.05). The MDA level increased in the Al only group in both the liver and brain (*p* < 0.05). Treatment with living or dead CCFM639 can reverse this parameter toward control levels (*p* < 0.05). However, it is worth noting that the living CCFM639 had a better protective effect than the dead CCFM639 on GSH, MDA, and SOD (*p* < 0.05).

### The BUN, CRE, ALT, and AST in serums

As shown in Table 3, the BUN level in serum was significantly elevated in the Al only group and was accompanied by increases in the CRE level and in ALT and AST activities (*p* < 0.05). Treatment with living or dead CCFM639 observably alleviated the alteration of these parameters (*p* < 0.05), with the exception of the BUN level.

**Table 3:** The influence of *L*. *plantarum* CCFM639 on Al-induced alterations of the enzyme activities of ALT and AST, and the levels of BUN and CRE in the serum.

Group	Mean level (/liter serum)		Mean activity (U/liter serum)	
	BUN (mmol)	CRE (μmol)	ALT	AST
Control	3.58 ± 0.24^a^	56.40 ± 1.80^a^	36.52 ± 0.85^a^	67.15 ± 1.86^a^
Al only	4.68 ± 0.32^b^	86.88 ± 2.94^b^	73.71 ± 2.23^b^	115.82 ± 3.54^b^
Al + living 639	3.94 ± 0.19^a^^b^	66.37 ± 2.86^a^^c^	46.15 ± 1.61^c^	86.08 ± 2.13^c^
Al + dead 639	4.02 ± 0.21^a^^b^	71.76 ± 3.52^c^	50.16 ± 0.73^c^	90.60 ± 1.73^c^

Data are mean ± SEM with ten mice in each group.

^a,b,c^The letters a, b and c indicate statistically significant differences (*p* < 0.05) among different groups.

### Morris water maze test

**Fig 7:**
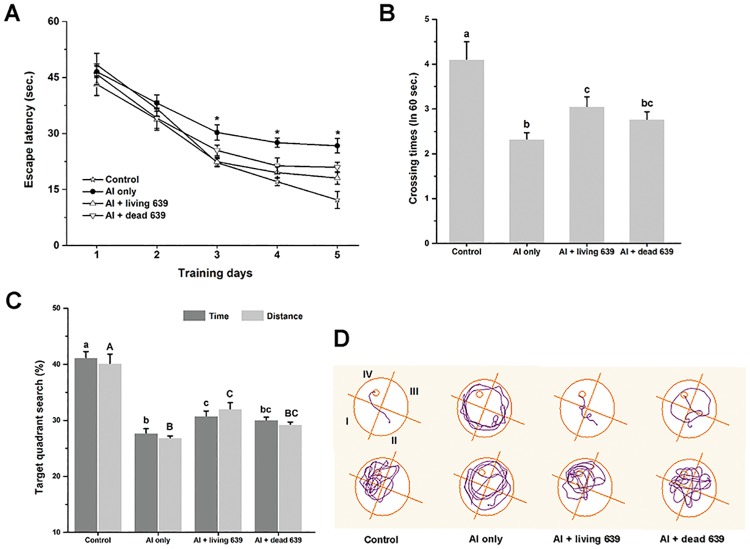
The influence of *L*. *plantarum* CCFM639 upon Al induced behavioral deficit (Morris water maze test). (A) Comparison of escape latency to platform during five training days (acquisition test). The escape latency is the time it takes to find the hidden platform. Asterisks above the lines show significant differences to the other three groups (*p* < 0.05). (B) Comparison of numbers of crossing over platform site on the sixth day (retrieval test). Lowercase letters on the top of the bars show significant differences among the four groups (*p* < 0.05). (C) Comparison of the time and distance travelled in the target quadrant with other quadrants on the sixth day. Uppercase and lowercase letters on the top of the bars show significant differences among the four groups (*p* < 0.05). (D) The moving trajectory of each group in the acquisition and retrieval test, the first four trajectories are in acquisition test, and the next four trajectories are in 60s retrieval test. 639 = *L*.*plantarum* CCFM639.

During the five training days, the escape latency of the mice in the Al + living or dead CCFM639 group showed greater improvement than those in the Al only group (Fig 7A). The mean escape latency of the Al only group was significantly higher than those of the other three groups after the second day (*p* < 0.05). Moreover, the platform crossing times and the distance travelled and time spent in the target quadrant were markedly lower for the Al only group than for the control and Al + living CCFM639 groups (*p* < 0.05). No significant differences were observed between the Al only and Al + dead CCFM639 groups (Fig 7B and 7C). The patterns of movement in Fig 7D show the trajectories followed by mice of different groups at the sixth day, which clearly show that the mice in the Al only group took more time to locate the hidden platform. In contrast, the mice in the Al + living CCFM639 group located the hidden platform more quickly, followed by those in the Al + dead CCFM639 groups. Moreover, when the platform was removed, the mice in the Al only group always swam along the wall, whereas those in the other three groups swam in the target quadrant. The performances of the mice in the Al + living CCFM639 group were better than those in the Al + dead CCFM639 group.

### Amyloid beta levels in the brains

**Fig 8:**
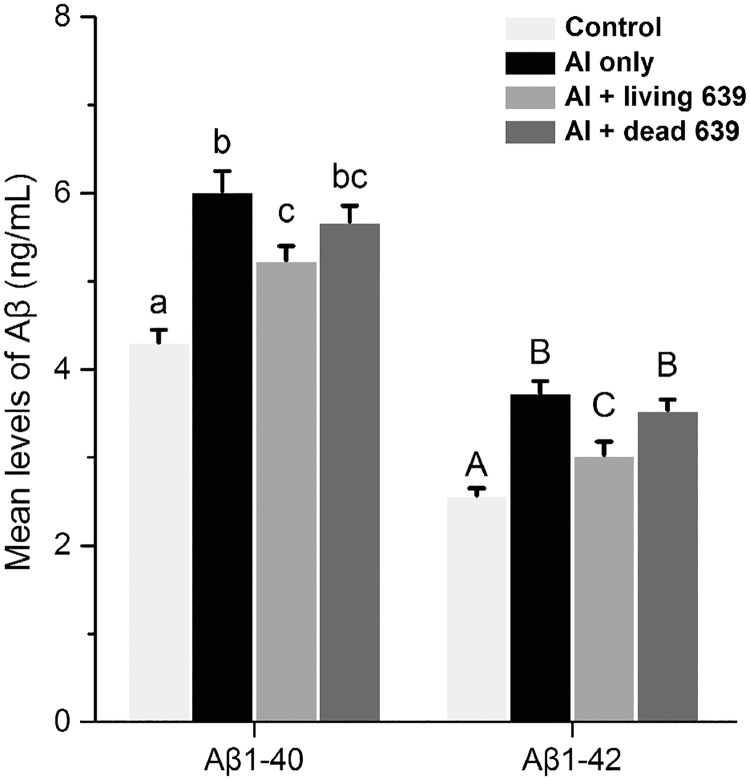
The influence of *L*. *plantarum* CCFM639 on Aβ levels in the brain of mice. Lowercases and uppercases on the top of the bars show significant differences among the four groups (*p* < 0.05).

The changes in the amyloid beta (Aβ) levels in the brain are presented in Fig 8. Al dramatically increased the levels of Aβ_1–40_ and Aβ_1–42_ in the brain (*p* < 0.05). After living CCFM639 treatment, the two parameters were significantly reduced (*p* < 0.05). No significant changes were observed between the Al only and Al + dead CCFM639 groups.

## Discussion

*Lactobacillus plantarum* CCFM639 was shown to alleviate acute Al toxicity in mice in our previous study [16]. It is important and necessary to investigate the protective abilities of *L*. *plantarum* CCFM639 against chronic Al toxicity because chronic Al exposure is a great threat to human and animal health [26]. To imitate chronic Al exposure, we orally introduced Al via drinking water, and the Al concentration was derived from previous studies [17]. Moreover, the intestinal Al sequestration ability of *L*. *plantarum* CCFM639 appeared to be important in previous studies because it can decrease Al absorption in the intestine, thereby reducing Al levels in other tissues. However, it was not clear whether the protective effects of this strain were a result of this protection route or any other routes. Therefore, in this study, we avoided the sequestration route by introducing Al into mice in first four weeks and then administered *L*. *plantarum* CCFM639, thus avoiding direct contact between Al and the strain in the intestine. After accumulating in the liver, a small amount of Al is re-excreted into the intestine through bile and then reabsorbed by enterocyte or excreted via the feces [2, 9]. It is thus possible that *L*. *plantarum* CCFM639 in the intestine may bind the re-excreted Al, which is then be excreted via the feces before intestinal re-absorption. This could account for the significant increase of fecal Al levels in the Al + living and dead CCFM639 group in the sixth week, even though there was no direct contact between Al and *L*. *plantarum* CCFM639 (Fig 3).

Several antioxidant enzymes, including CAT, SOD and GPx are crucial in the cellular defense against ROS and free radicals [27]. Previous research showed that Al exposure led to oxidative stress with a decrease in SOD, GPx and CAT activity and the GSH level, and a higher level of MDA [28, 29]. One proposed mechanism by which Al modifies the activities of antioxidant enzymes is its capacity to interact with essential trace metals [30–32]. Mg, Zn and Cu are the cofactors of SOD. Cu overload causes obvious oxidative stress damage [33]. Chronic Cu overload would cause Fe overload, it results increases in AST and ALT levels, as well as in MDA content [34]. Studies have shown that appropriate supplementation of Zn was effective in alleviating tissue damage caused by Al exposure, possibly due to activation of Zn-SOD, which decreases Al induced oxidative stress injury [35]. Imbalances in Al, Fe, Cu and Zn levels caused by Al overload can lead to obvious oxidative stress in liver, which further results in damage to hepatic cells and liver dysfunction [36]. Probiotic can alleviate Al toxicity via many mechanisms such as reducing oxidative stress, inhibiting NO synthase-2 expression and so on [37, 38]. When NO is produced in excess, it causes deleterious effect indirectly through the creation of reactive nitric oxygen species (RNOS), responsible for the oxidative stress. NO has been implicated as a pathogenic mediator in a variety of conditions, such as Alzheimer’ diseases [39, 40]. The possible antioxidative mechanisms of LAB may involve scavenging of ROS, possessing reducing activity and regulating trace elements [12]. It is noticeable that *L*. *plantarum* CCFM639 elevated the GPx, SOD, and CAT activity; decreased the MDA level, and increased GSH level, which are associated with antioxidant defense systems. Living *L*. *plantarum* CCFM639 had a greater antioxidative effect than the dead strain. One reason may be that the living strain can improve the absorption and bioavailability of trace elements [41–43]. Moreover, AST, ALT, CRE and BUN, the biomarkers of hepatic and renal injuries [29, 44], were also alleviated by living and dead CCFM639.

There is increasing evidence to indicate that Al is a neurotoxic metal and may cause learning and memory impairment [23, 45]. Al expsoure leads to amyloid beta (Aβ) peptide accumulation in the brain. Increased Aβ can induce ROS generation, and ROS causes oxidative stress and thus deteriorates the learning ablility and cognitive function [10, 46, 47]. Many studies indicate that oxidative stress is an important factor in the development and progression of Alzheimer’ diseases [48, 49]. Mounting evidence suggests that ingestion of probiotics, including *Lactobacillus* and *Bifidobacteria*, increases memory function and improves cognition via the gut-brain axis [50–53]. The Morris water maze is a useful tool for evaluation of learning and memory in mice [54]. Our results in this study demonstrate that *L*. *plantarum* CCFM639 can alleviate cerebral oxidative stress in the mice. The alleviations subsequently improved memory deficits of Al-exposed mice and decreased Aβ accumulation in the brain. These results are consistent with recent findings confirming that probiotics supplementation may affect physiology and behavior in both diseased and healthy states [55].

**Fig 9:**
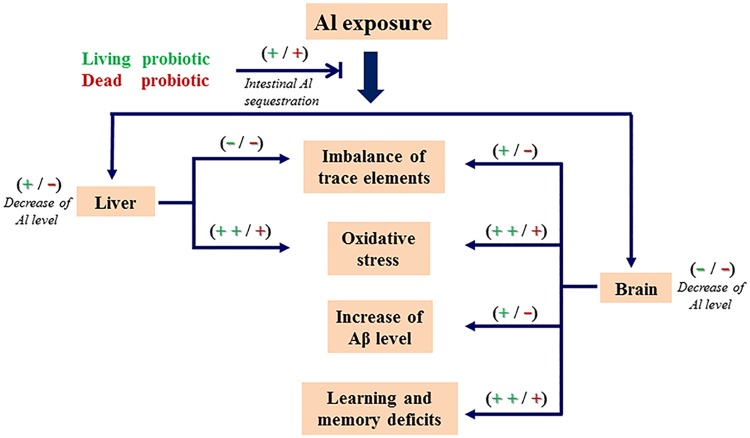
The potential protection mechanism of *L*. *plantarum* CCFM639 against Al-induced liver and brain injuries.

In addition, the differences between mice in the Al + living and the dead CCFM639 groups provide insight into the protection of *L*. *plantarum* CCFM639 against chronic Al toxicity. Taking all effects of fecal Al excretion, tissue Al accumulation, and oxidative stress into consideration, living CCFM639 treatment offers the best protection against chronic Al toxicity. Living CCFM639 can stimulate intestinal peristalsis, increase the absorption of essential elements and alleviate oxidative stress directly, whereas the dead strain may not have these properties. Therefore, our results show that living and dead CCFM639 strains have similar Al binding ability *in vitro*, but living strain treatment had more protective effects than dead strain treatment in animal experiments (Fig 9).

## Conclusion

Living and dead *L*. *plantarum* CCFM639 treatment offers direct protection against chronic Al toxicity by alleviating oxidative stress besides intestinal Al sequestration, and living *L*. *plantarum* CCFM639 provides better protection than the dead strain. *L*. *plantarum* CCFM639 thus has potential to be a supplementary dietary ingredient for alleviating chronic Al toxicity.
